# Supplementary material for: Nintedanib-αVβ6 Integrin Ligand Conjugates Reduce TGFβ-Induced EMT in Human Non-Small Cell Lung Cancer
Source: Int J Mol Sci. 2023 Jan 12;24(2):1475. doi: 10.3390/ijms24021475 (PMC9861180; doi:10.3390/ijms24021475)
Supplement: Supplementary file 1 [file ijms-24-01475-s001.zip › ijms-2065441-supplementary.pdf]

Supplementary material:

Nintedanib-αVβ6 Integrin Ligand Conjugates reduce TGFβ-induced EMT in human non-small cell lung cancer

Elena Andreucci[1], Kelly Bugatti[2], Silvia Peppicelli[1], Jessica Ruzzolini[1], Matteo Lulli[1], Lido Calorini[1], Lucia Battistini[2], Franca Zanardi[2], Andrea Sartori[2*], Francesca Bianchini*[1]

[1] Department of Experimental and Clinical Biomedical Sciences "Mario Serio", University of Florence, Viale Morgagni 50, 50134, Florence, Italy
[2] Department of Food and Drug, University of Parma, Parco Area delle Scienze 27A, 43124 Parma, Italy
* Correspondence: francesca.bianchini@unifi.it, andrea.sartori@unipr.it.

Table of contents:

1. **Synthesis of the conjugated compounds** ______ p. S2
2. **Expression of $\alpha v\beta 3$ in A549 and H1975 human NSCLC cells grown in the presence or in the absence of TGFβ, and inhibition of adhesion of A549 on vitronectin substrate** ______ p. S4
3. **Expression of $\alpha v\beta 6$ integrin receptor in K562 human erythroleukemic cells and internalization of conjugated compounds or nintedanib** ______ p. S5
4. **Expression of $\alpha v\beta 6$ integrin receptor in SSM2c human melanoma cells and internalization of conjugated compounds or nintedanib** ______ p. S5
5. **Expression of $\alpha v\beta 6$ integrin receptor in L929 murine fibroblasts and internalization of conjugated compounds or nintedanib** ______ p. S6
6. **Dose-dependent A549 and A549/TGFβ-treated cell internalization of conjugated compounds or nintedanib** ______ p. S6
7. **Inhibition of TGFβ-treated A549 cell proliferation** ______ p. S7
8. **Estimated enzyme activity of nintedanib and compound 1** ______ p. S7
9. **Inhibition of L929 spheroid growth** ______ p. S8

1. Synthesis of the conjugated compounds

CHEMISTRY. ***General.*** All chemicals were of the highest commercially available quality and were used without other purification. Automated flash column chromatography was performed with the Biotage Isolera One system using KP-C18-HS cartridges (reverse phase). HPLC purifications were carry out on a Prostar 210 apparatus (UV detection) using C18-10 μm columns (Discovery BIO Wide Pore 10 × 250 mm). Purity of the final compounds was checked by HPLC and was >99%. ESI-mass spectra were collected on UHPLC/ESI-MS system (ACQUITY Ultra Performance LC; ESI, positive ions, Single Quadrupole analyzer) and are reported in the form of (*m*/*z*). High resolution mass analysis was performed on LTQ ORBITRAP XL Thermo apparatus. NMR spectra were recorded on AV400 (Bruker) spectrometer. Chemical shifts (*δ*) are reported in ppm. Multiplicities of signals are reported as s (singlet), d (doublet), t (triplet), q (quartet), m (multiplet), and b (broad). Coupling constants, *J*, are in Hertz. ^{1}H assignments are corroborated by ^{1}H-^{1}H COSY and ^{1}H-^{1}H TOCSY.

Abbreviations. Amp, *cis*-4-amino-L-proline; DMF, *N*,*N*-dimethylformamide; Pmc, 2,2,5,7,8-pentamethylchroman-6-sulfonyl; TFA, trifluoroacetic acid; TIS, triisopropylsilane.

Materials. Trifluoroacetic acid and triisopropylsilane were commercially available and were used as such without further purification. Compound **5** [32] and intermediates **6**-**8** [26] were prepared according to reported procedures.

General method for HPLC purification. The final conjugates were purified by reverse phase HPLC equipped with a preparative column (C18-10 μm, 21.2 x 250 mm column), with the solvent system H_2O + 0.1% TFA (Solvent A) and ACN (solvent B), using a linear gradient from 5% B to 50% B over 23 min, 50% B for 3 min, from 50% B to 5% B over 3 min, (flow rate 8.0 mL/min; detection at 220 nm).

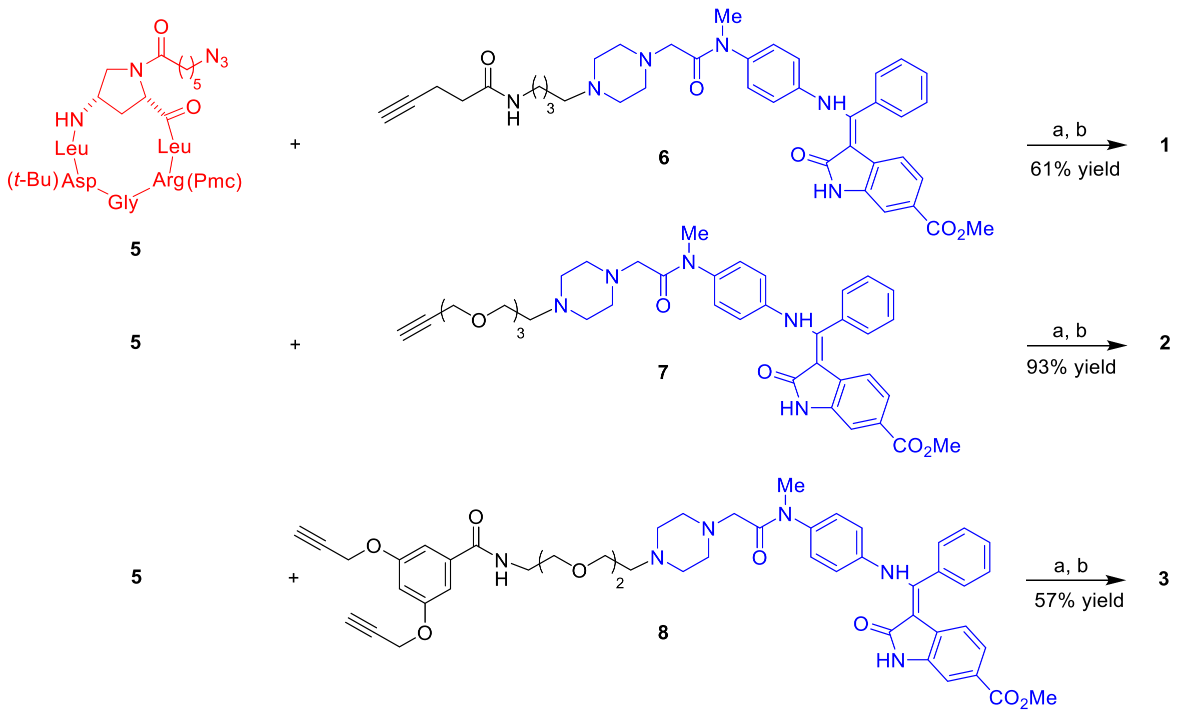

Figure S1. Synthesis of covalent conjugates **1**-**3**. Reagents and conditions: (a) $Cu(OAc)_2$ 0.3 equiv, sodium ascorbate 0.6 equiv, DMF/H_2O 3:7, rt, 6.5-14 h; (b) TFA/TIS/H_2O 95:2.5:2.5, rt, 1 h

Synthesis of compound 1. To a solution of compound **6** (7.4 mg, 0.011 mmol, 1 eq) and cyclopeptide **5** (15.0 mg, 0.012 mmol, 1.1 eq) in DMF (1.6 mL), 0.67 mL of a water solution of $Cu(OAc)_2$ (0.64 mg, 0.003 mmol, 0.3

eq) and sodium ascorbate (1.3 mg, 0.007 mmol, 0.6 eq) were added. After 3 cycles of argon/vacuum, the reaction was left under stirring under argon atmosphere. After 6.5 h, the solvent was removed under reduced pressure and the residue was washed with water (3x) and diethyl ether (3x). The protected intermediate was checked by MS analysis (MS (ES^+) *m/z* 1805.0 $[M+H]^+$) and then it was deprotected using a solution of TFA:TIS:H_2O 95:2.5:2.5 (0.55 mL). The reaction was kept under stirring for 1 h, then the solvent was removed under reduced pressure and the crude was purified by reverse phase HPLC, using the described general method (R_t = 23.0 min), giving the final conjugate **1** (12.3 mg, 61% yield). ^1H NMR (400 MHz, CD_3OD) *δ* 7.66 (s, 1H, CH triazole), 7.54-7.56 (m, 5H, ArH), 7.52 (m, 2H, ArH), 7.30 (dd, *J* = 8.3, 1.5 Hz, 1H, ArH), 7.16 (m, 2H, ArH), 6.96 (m, 2H, ArH), 6.85 (m, 2H, ArH), 5.97 (d, *J* = 8.3 Hz, 1H, ArH), 4.39-4.31 (m, 6H, αAsp+αLeu+H2Amp+CH_2), 4.21 (bt, *J* = 6.6 Hz, 1H, H4Amp), 4.15-4.07 (m, 2H, αGly+αArg), 3.89 (m, 1H, H5Amp), 3.87 (m, 3H, CH_3), 3.78 (m, 1H, αGly), 3.37 (m, 1H, H5Amp), 3.29-3.17 (m, 10H, δArg+CH_2), 3.12 (m, 4H, CH_2), 3.01 (bt, *J* = 6.9 Hz, 2H, CH_2), 2.92 (dd, *J* = 17.6, 4.6 Hz, 1H, βAsp), 2.88-2.75 (m, 2H, βAsp+H3Amp), 2.56 (m, 2H, CH_2), 2.33 (m, 2H, CH_2), 1.89 (m, 4H, CH_2), 1.79-1.47 (m, 15H, βArg+γArg+γLeu+βLeu+CH_2+H3Amp), 1.31 (m, 4H, CH_2), 0.98 (dd, *J* = 17.9, 6.5 Hz, 6H, δLeu), 0.93 (dd, *J* = 17.9, 6.5 Hz, 6H, δLeu). HRMS(ES+) $C_{74}H_{103}N_{19}O_{14}$ calcd for $[M+3H]^{3+}$ 494.9389, found 494.9392.

Synthesis of compound 2. Dual conjugate **2** was synthesized as described for compound **1**, starting from compound **7** (9.1 mg, 0.013 mmol, 1.0 eq) and cyclopeptide **5** (18.0 mg, 0.014 mmol, 1.1 eq). The protected intermediate was checked by MS analysis (MS (ES^+) *m/z* 1823.9 $[M+H]^+$) and, after 8 h, it was deprotected using a solution of TFA:TIS:H_2O 95:2.5:2.5 (0.65 mL). The reaction was kept under stirring for 1 h, then the solvent was removed under reduced pressure and the crude was purified by reverse phase HPLC, using the described general method (R_t = 23.2 min), giving the final conjugate **2** (19.5 mg, 93% yield). ^1H NMR (400 MHz, CD_3OD) *δ* 8.00 (s, 1H, CH triazole), 7.79-7.57 (m, 5H, ArH), 7.52 (m, 2H, ArH), 7.30 (dd, *J* = 8.4, 1.6 Hz, 1H, ArH), 7.17 (m, 2H, ArH), 6.95 (m, 2H, ArH), 5.97 (d, *J* = 8.4 Hz, 1H, ArH), 4.61 (s, 2H, O-CH_2-triazole), 4.50-4.36 (m, 6H, αAsp+αLeu+CH_2+ H4Amp), 4.21 (m, 1H, αArg), 4.11 (m, 2H, αGly+H2Amp), 3.91-3.78 (m, 7H, CH_2+αGly+ H5Amp+CH_2), 3.66 (m, 10H, CH_2), 3.38 (m, 1H, H5Amp), 3.26-3.15 (m, 8H, βArg+CH_2), 3.12 (m, 2H, CH_2), 2.95 (dd, *J* = 17.5, 4.5 Hz, 1H, βAsp), 2.86-2.76 (m, 3H, βAsp+CH_2), 2.55 (m, 1H, H3Amp), 2.42-2.25 (m, 2H, CH_2), 1.97-1.57 (m, 17H, βArg+γArg+γLeu+ βγLeu+CH_2+H3Amp), 1.33 (m, 4H, CH_2), 0.98 (dd, *J* = 18.2, 6.1 Hz, 6H, δLeu), 0.93 (dd, *J* = 18.2, 6.1 Hz, 6H, δLeu). HRMS(ES+) $C_{74}H_{104}N_{18}O_{16}$ calcd for $[M+3H]^{3+}$ 501.2704, found 501.2709.

Synthesis of compound 3. Covalent conjugate **3** was synthesized as described for compound **1**, starting from compound **8** (10.0 mg, 0.012 mmol, 1 eq) and cyclopeptide **5** (32.4 mg, 0.0265 mmol, 2.3 eq) in DMF (1.69 mL). The reaction was quenched after 16 h, and the protected intermediate was checked by MS analysis (MS (ES^+) *m/z* 3124.5 $[M+H]^+$) and then it was deprotected using a solution of TFA:TIS:H_2O 95:2.5:2.5 (0.57 mL). The reaction was kept under stirring for 1 h, then the solvent was removed under reduced pressure, and the crude was purified by reverse phase HPLC, using the described general method with the only exception of using column B with flow rate 3.0 mL/min (R_t = 23.1 min), giving the final conjugate **3** (16.6 mg, 57% yield). ^1H NMR (400 MHz, CD_3OD) *δ* 8.10 (bs, 2H, CH triazole), 7.66-7.55 (m, 5H, ArH), 7.50 (m, 2H, ArH), 7.30 (m, 1H, ArH), 7.12 (m, 4H, ArH), 6.90 (m, 2H, ArH), 5.96 (d, *J* = 8.1 Hz, 1H, ArH), 5.20 (bs, 4H, ArO-CH_2-triazole), 4.51-4.36 (m, 10H, αAsp+αLeu+H4Amp+H2Amp), 4.19 (m, 2H, αArg), 4.10 (m, 4H, CH_2), 3.89-3.84 (m, 5H, CH_3+αGly), 3.83-3.75 (m, 4H, αGly+H5Amp), 3.68 (m, 8H, CH_2), 3.58 (m, 2H, CH_2), 3.42 (m, 2H, H5Amp), 3.30-3.13 (m, 10H, δArg+CH_2), 3.98-3.81 (m, 8H, βAsp+CH_2), 2.55 (m, 2H, H3Amp), 2.42-2.20 (m, 4H, CH_2), 1.98-1.83 (m, 10H, H3Amp+CH_2), 1.80-1.54 (m, 22H, βLeu+βArg+γArg+CH_2), 1.36-1.26 (m, 8H, CH_2+γLeu), 0.97 (dd, *J* = 18.4, 6.1 Hz, 12H, δLeu), 0.92 (dd, *J* = 18.4, 6.1 Hz, 12H, δLeu). HRMS(ES+) $C_{119}H_{171}N_{32}O_{27}$ calcd for $[M+3H]^{3+}$ 827.7742, found 827.7717.

2. Expression of $\alpha v\beta 3$ in A549 and H1975 human NSCLC cells grown in the presence or in the absence of TGFβ, and inhibition of adhesion of A549 on vitronectin substrate

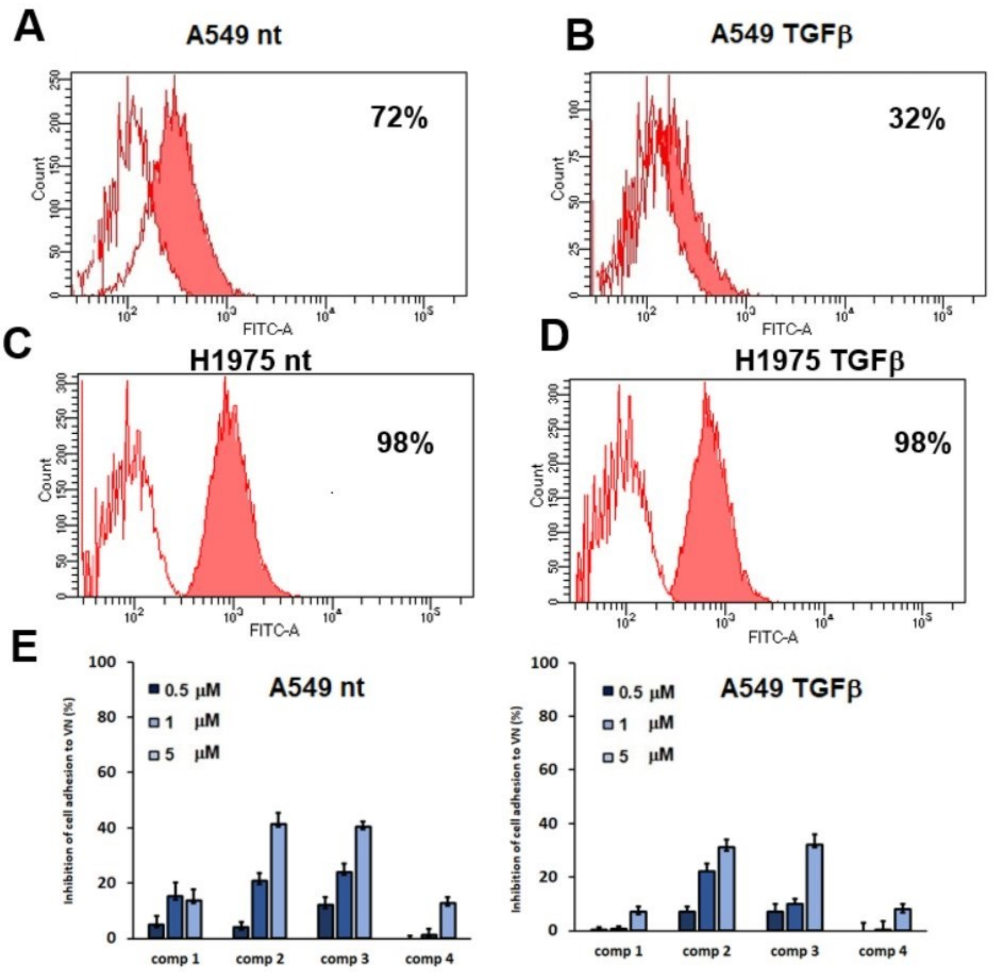

Figure S2. Expression of $\alpha v\beta 3$ integrin receptor in A549 and H1975 human NSCLC cells grown for 48h in the absence (A, C) and in the presence (B, D) of TGFβ (10 ng/ml). Cells were stained with rabbit anti-human αvβ3 antibody followed by FITC-conjugated anti-rabbit immunoglobulin (full histograms). As a negative control (open histogram), cells were stained in only with FITC-conjugated anti-rabbit immunoglobulin. (E) Inhibition of A549, and TGFβ-treated A549 cell adhesion to VN (5 μg/mL) in the presence of compounds **1**-**3**, or *c*(AmpLRGD) **4** (2 h). The inhibitory activity was calculated as the percentage of cell adhesion to VN in untreated cells and was expressed as means ± SEM. Experiments were carried out in triplicate.

3. **Expression of $\alpha v\beta 6$ integrin receptor in K562 human erythroleukemic cells and internalization of conjugated compounds or nintedanib**

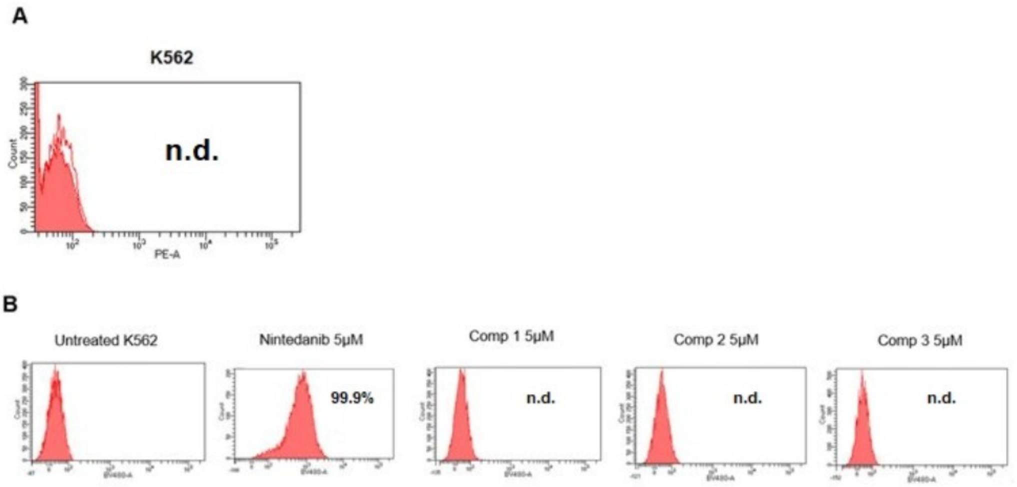

Figure S3. Expression of $\alpha v\beta 6$ integrin receptor in K562 human erythroleukemia cells (A). Cells were stained with rabbit anti-human αvβ6 antibody followed by FITC-conjugated anti-rabbit immunoglobulin (full histograms). As a negative control (open histogram), cells were stained only with FITC-conjugated anti-rabbit immunoglobulin. (B) Representative images of internalization of conjugates **1**-**3** or nintedanib as assessed by flow cytometry measurements at 5 μM concentration.

4. **Expression of $\alpha v\beta 6$ integrin receptor in SSM2c human melanoma cells and internalization of conjugated compounds or nintedanib**

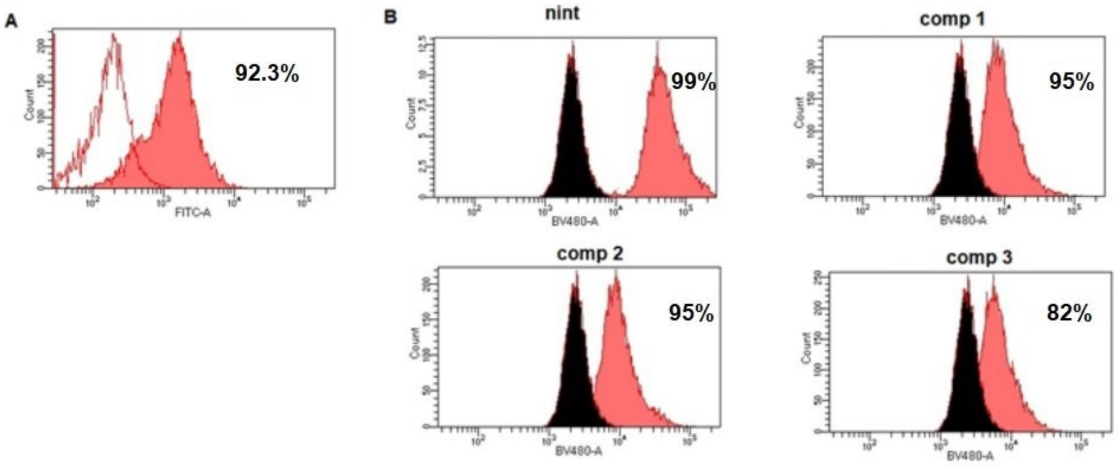

Figure S4. (A) Expression of $\alpha v\beta 6$ integrin receptor in SSM2c human melanoma cells. Cells were stained with mouse anti-human αvβ6 antibody followed by FITC-conjugated anti-mouse immunoglobulin (full histograms). As a negative control (open histogram), cells were stained only with FITC-conjugated anti-mouse immunoglobulin. (B) Representative images of SSM2c internalization of conjugates **1**-**3** or nintedanib as assessed by flow cytometry. (FacScan FLT1/BV405ex480em-A). Cells were exposed for 24 h to nintedanib or conjugates **1**-**3** at 5 μM concentration.

5. Expression of $\alpha v\beta 6$ integrin receptor in L929 murine fibroblasts and internalization of conjugated compounds or nintedanib

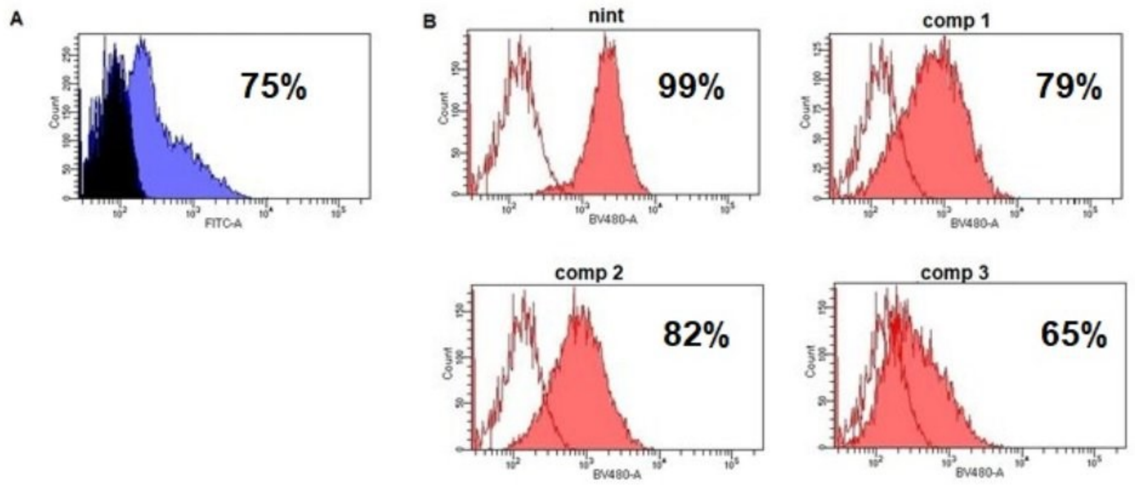

Figure S5. (A) Expression of αvβ6 integrin receptor in L929 murine fibroblasts. Cells were stained with mouse αvβ6 antibody followed by FITC-conjugated anti-mouse immunoglobulin (blue histograms). As a negative control (black histogram), cells were stained in only with FITC-conjugated anti-mouse immunoglobulin. (B) Representative images of L929 internalization of conjugates **1**-**3** or nintedanib as assessed by flow cytometry. (FacScan FLT1/BV405ex480em-A). Cells were exposed for 24 h to nintedanib or conjugates **1**-**3** at 5 μM concentration.

6. Dose-dependent A549 and A549/TGFβ-treated cell internalization of conjugated compounds or nintedanib

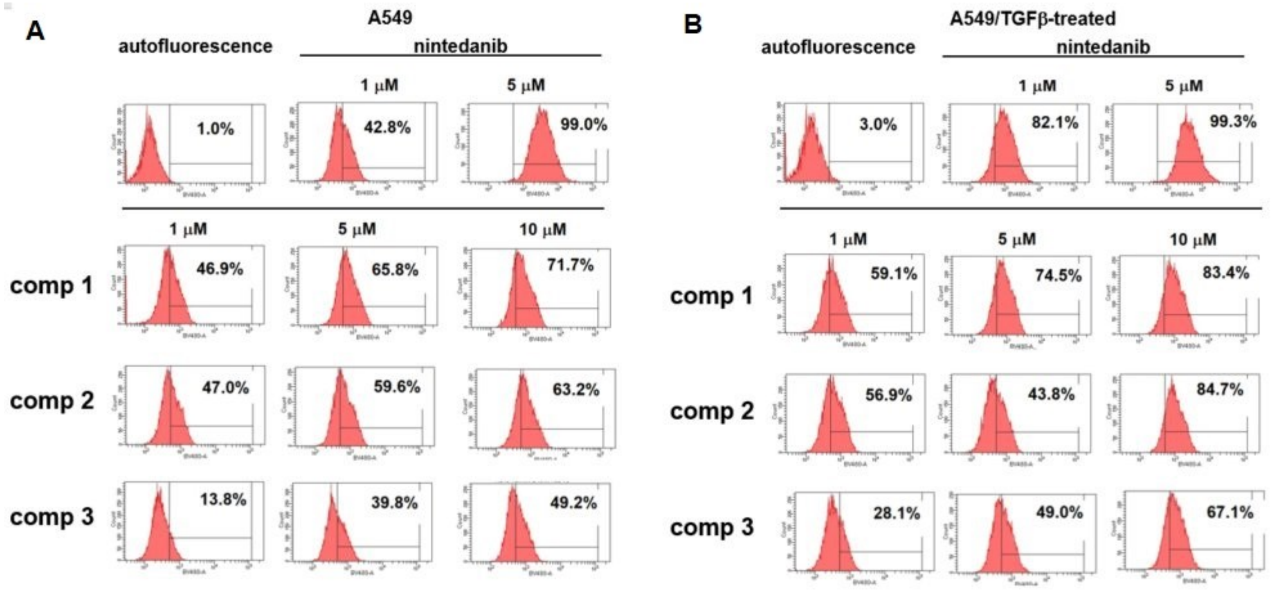

Figure S6. Representative images of internalization of conjugates **1**-**3** or nintedanib as assessed by flow cytometry measurements. Fluorescence intensity (FacScan FLT1/BV405ex480em-A) in A549 cells (A) and TGFβ-treated A549 cells (B) exposed for 24 h to nintedanib or conjugates **1**-**3** at different concentrations (1-10 μM). Percentages indicate fluorescence (BV480-A)-positive cells from three independent experiments.

The treatment with TGFβ induced a shift to the right of the cell population distribution except for those cells exposed to nintedanib. The percentages of positive population of cells, exposed to compound **1** (1 μM) were

46.9% and 59.1%, for untreated and TGFβ-treated cells, respectively. These percentages increased after the exposure to 10 μM concentration (71.7% and 83.4%, respectively). The percentages of internalization in cells exposed to compound **2** (1 μM) were 47.0% and 56.9% for untreated and TGFβ-treated cells, respectively. After the exposure to 10 μM concentration of **2**, the percentages rose to 63.2% and 84.7%. Interestingly, internalization of compound **3** was lower with respect to both compounds **1** and **2**, even at 10 μM concentration, we found a 49.2% internalization in untreated cells and 67.1% in cells exposed to TGFβ. The percentages of internalization of compounds **1** and **2** (at 10 μM) in TGFβ activated cells were close to those of cells exposed to nintedanib 5 μM (Figure S6).

7. Estimated enzyme activity of nintedanib and compound 1

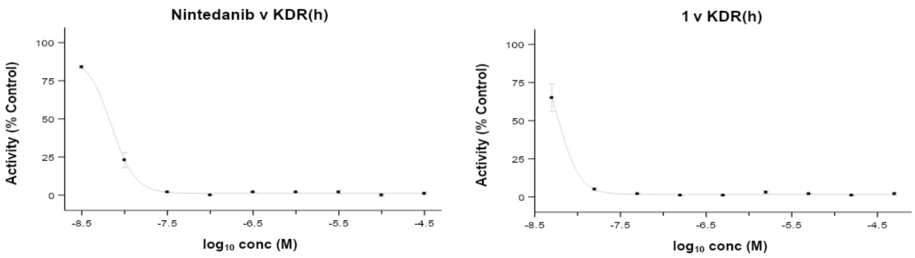

Figure S7. Estimated IC_{50} values of nintedanib and compound **1** were obtained from Eurofins enzyme profiling services KinaseProfiler™, IC 50 Profiler™. Work order: FR095-0025710.

8. Inhibition of cell proliferation

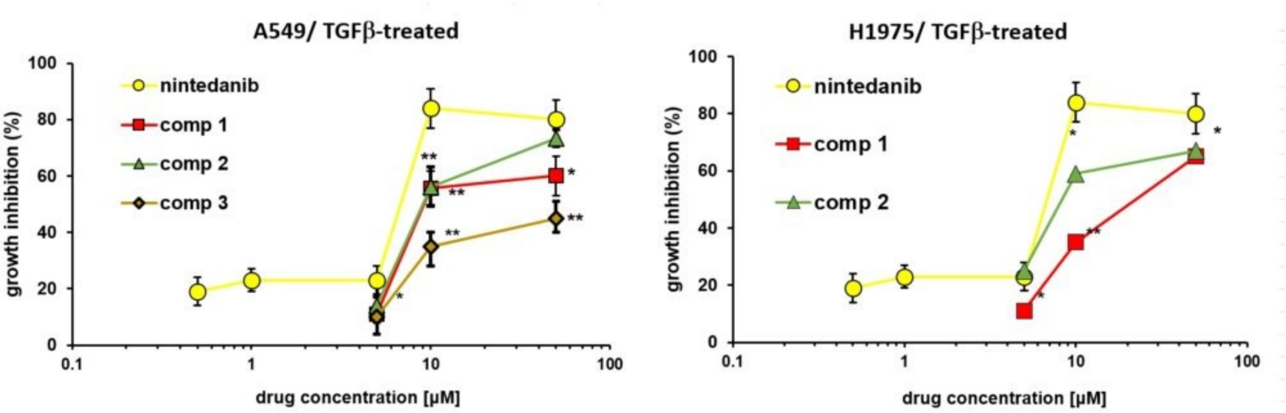

Figure S8. MTT assay of TGF*β*- treated A549 cells (left panel) and TGF*β*-treated H1975 cells (right panel) exposed for 24 h to different concentrations of compounds **1**-**3** or nintedanib (0.5 μM, 1 μM, 5 μM, 10 μM, 50 μM). Data are expressed as mean ± SEM of the percentage of viability and are representative of three independent experiments (n=4). *p<0.0 1 and **p<0.001 vs equimolar concentration of nintedanib by One-way ANOVA followed by Tukey's multiple comparison test.

While nintedanib inhibited cell proliferation more efficiently than conjugates **1** and **2**, at 10 μM, the inhibitory activity of compound **2** approached that of nintedanib only at 50 μM concentration. Compound **3** behaved less efficiently than the other two conjugates revealing only a weak inhibitory activity even at 10 μM.

9. Inhibition of L929 spheroid growth

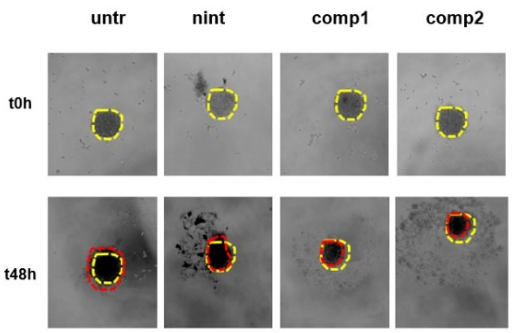

Figure S9. Representative images of L929 spheroid growth inhibition after 48 h exposure to nintedanib, compound **1**, or **2**.
